# The magnetic resonance imaging evaluation of condylar new bone remodeling after Yang’s TMJ arthroscopic surgery

**DOI:** 10.1038/s41598-021-84591-1

**Published:** 2021-03-04

**Authors:** Minjun Dong, Zixian Jiao, Qi Sun, Xiaofeng Tao, Chi Yang, Weiliu Qiu

**Affiliations:** 1 Department of Radiology, Shanghai Ninth People’s Hospital, Shanghai Jiaotong University School of Medicine, Shanghai, 200011 China; 2 Department of Oral Surgery, Shanghai Ninth People’s Hospital, Shanghai Jiaotong University School of Medicine, Shanghai, 200011 China; 3 Department of Oral Maxillofacial Surgery, Shanghai Ninth People’s Hospital, Shanghai Jiaotong University School of Medicine, Shanghai, 200011 China

**Keywords:** Diseases, Medical research

## Abstract

To evaluate the post-operative condylar bone remodeling after the treatment of Yang’s arthroscopic surgery. Consecutive cases from Jan 2017 to May 2018 that received Yang’s arthroscopic surgery were included in this study, the TMJ MRI examinations were performed preoperatively and postoperatively (follow up for 1 year or more), and condylar bone remodeling was estimated. A total of 229 patients (29 male and 200 female) were included in the study, 161 patients had new bone formation, and the average age was 17.5 ± 2.1a. There was no new bone formation in 68 patients with an average age of 24.5 ± 0.7a. The percentage of new bone formation patients in 10–15 years of age was 94.33% and decreases as the age increases. In the position of new bone formation, the posterior slope of condyle was the most (129 joints), the second was the top of condyle (54 joints), the third was around condyle (33 joints), only 25 joints had new bone on the anterior slope of condyle. After TMJ arthroscopic surgery, the condyle has the ability to form new bone, and the younger the age, the stronger the ability of new bone formation. The formation of new bone was most in posterior slope and least in anterior slope of condyle.

## Introduction

Temporomandibular joint (TMJ) anterior disc displacement (ADD) is one of the most common types of the TMJ diseases, including anterior disc displacement with reduction (ADDWR) and anterior disc displacement without reduction (ADDWoR). In adolescent patients, the unilateral ADD will lead to mandible asymmetry^1,2^, and if left untreated, the mandible height would decrease during its natural course, which would result in more severe facial asymmetry^3,4^. Bilateral simultaneous onset will lead to mandibular retrusion. In recent years, Yang^5,6^ introduced a new arthroscopic disc repositioning and suturing technique for treating TMJ ADD. After years of clinical application, it is reported that the clinical effect of this operation is very promising. However, there are few studies on the remodeling of condylar bone after arthroscopic surgery. Whether the condylar bone degenerative changes will be intensified or stopped and whether the condylar bone growth potential will be restored still remains unclear.

Compared with other imaging methods, such as X-ray, computed tomography (CT), and cone beam computed tomography (CBCT), magnetic resonance imaging (MRI) can clearly display various anatomical structures around the joint, including bone, cartilage, articular disc, masticatory muscles, and synovial fluid. Therefore, as a noninvasive examination, MRI is considered suitable for analyzing the TMJ disc and surrounding anatomical structures in normal and diseased state^7^.

Thus, this study was aimed at estimating the condylar bone remodeling after arthroscopic surgery using TMJ MRI.

## Patients and methods

### Study design and samples

This longitudinal retrospective study was approved by the institutional review board of Shanghai Ninth People’s Hospital, Shanghai Jiao Tong University School of Medicine. All methods were performed in accordance with the relevant guidelines and regulations. The study sample was derived from the patients seeking for treatment in the TMJ division of the Department of Oral Surgery in Shanghai Ninth People’s Hospital affiliated to Shanghai Jiao Tong University School of Medicine from Jan 2017 to May 2018. Patients included in the study are aware of the study and sign the informed consent. All the pre-operative and post-operative MRI examinations were taken in the Department of Radiology. The images of the TMJ MRI were evaluated by the same experienced radiologists.

Patients included in this study,

(1) had MRI examination on the first visit and diagnosed as ADD (both ADDWR and ADDWoR); (2) received TMJ arthroscopic surgery in Department of Oral Surgery and the surgeries were performed by the same surgeon; (3) had MRI examination post-operatively 1 year or more follow-up and the disc was in a satisfactory position; (4) had no medicine administration before or after the surgery and during the follow-up period; (5) were on a regular follow-up in the authors’ department; (6) had no history of infection, injuries to the jaws, or congenital, developmental, and systematic disorders that could affect craniofacial growth.

Patients excluded from this study (1) had less than 2 MRI scans owing to the patients no show during follow-up or had MRI examinations in other hospital; (2) had relapse which the disc moved anteriorly again.

### Participants' statement

Patients included in this study with age under 18 have signed the informed consent obtained from a parent and/or legal guardian. The statement reads as follows.

*“The study doctor has explained to me in detail the purpose and process of the trial, as well as the possible risks and benefits of participating in the trial. I have read the instructions carefully and have enough time to ask questions. At present, I have no questions.*

*I am willing to participate in this test, and I can withdraw from the test at any time for any reason without any loss. I will follow the research doctor's instructions during the trial. If any adverse events occur during the trial, I will immediately notify the clinical trial physician or other researchers. If this test causes any obvious damage to my health (as mentioned in the test plan and instructions), I will receive active treatment.*

*I know that if I use other drugs myself without discussing with the research doctor in advance, and if I have any problems with my health, I will not inform my research doctor in time, which will affect my guarantee in the trial.*

*I agree that the data obtained from this clinical trial can be used for recording, storing and processing in a computer. In addition, I agree that the researchers, supervisors, inspectors, ethics committee and inspectors of drug supervision and administration departments related to this trial can consult my case records on the premise of following the principle of confidentiality. I understand that these records are being reviewed to ensure that the information collected from this trial is true, accurate and reliable.*

*In conclusion, I agree to participate in this clinical trial, and I have obtained a copy of this signed informed consent.”*

### Evaluation of TMJ MRI

MRI scans were obtained using a 3.0 T MR scanner (Ingenia, Philips Healthcare Systems, The Netherlands) with a 6-channel dS Flex M surface coil receiver. Details about the TMJ MRI and obtained in the clinic were the same as follows: proton density-weighted imaging (PDWI) sequences were acquired in oblique sagittal planes with closed mouth; T_2_-weighted images (T_2_WI) were acquired in oblique coronal planes with closed mouth; and T_2_WI were acquired in oblique sagittal planes with open mouth (with auxiliary opening fixation device). The main scanning parameters of sequences are shown in Table 1.

**Table 1:** Acquisition parameters of the applied MR sequences.

Sequences	Position	TR/TE (ms)	FOV (mm)	Reconstruction matrix	Thickness/gap (mm)	Flip angle (°)	Slices	NSA	TA	Bandwidth (kHz)
T_2_WI	Closed (OCor)	2500/70	110 × 110	384 × 224	1.5	90	16	2	2′1″	290.7
T_2_WI	Opened (OSag)	2500/65	110 × 110	384 × 224	2	90	16	2	2′07″	206.5
PDWI	Closed (OSag)	2000/20	110 × 110	400 × 256	2	90	16	2	2′05″	234.8

Qualitative evaluation of postoperative MRI was performed by the same two experienced radiologists. Both observers assessed the images together and expressed the same opinion. The evaluation included the disc position (close-mouth and open-mouth position) and condylar morphology. The condylar morphology evaluation mainly focused on the new bone formation which was identified when high signal intensity around or above the original condylar cortical bone was seen and continuous or discontinuous low signal cortical bone images are visible on the periphery of high-signal images. The condylar new bone formation was recorded as present or absent.

### Statistical analysis

All data were expressed as the mean ± SD. Results were analyzed with Analysis of Student T test using SPSS 13.0 software (SPSS Inc., USA). *p* < 0.05 indicated a significant difference between the groups.

## Results

### Basic description of the patients’ group

From Jan 2017 to May 2018, 229 patients of 335 joints received arthroscopic surgery for treating ADDWoR. Among the patient group, there are 29 (12.5%) male patients and 200 (86.5%) female patients. The average age of the patients is 21.5 ± 3.5a (ranging from 10 to 62a.). All the MRI images of the patients were clear and satisfactory.

The patients were then divided into 5 groups based on the age differentiation for further analysis. (1) Age of 10–15 group, (2) age of 16–20 group, (3) age of 21–25 group, (4) age of 26–30 group, (5) age above 30 group.

### Gender and age distribution of the patients’ group

The new bone formation of the condyle was found in 161 (70.3%) patients. There were 26 (11.4%) male patients and 135 (58.9%) female patients, with an average age of 17.5 ± 2.1a. However, there were 68 (29.7%) patients that had no condylar bone remodeling, including 3 (1.3%) male patients and 65 (28.4%) female patients with an average age of 24.5 ± 0.7a. There was significant difference in age distribution between the group with new bone formation and the group without new bone formation (*p < 0.05).

By grouping the gender of the patients, in the male group, there were 26 out of 29 patients that have new bone formation, with the percentage of 89.66%. While in the female group, there were 135 out of 200 patients that have new bone formation, with the percentage of 67.5%.

Further analysis of the correlation between patients’ age and new bone formation, the results showed that in the age of 10–15 group, there were 50 out of 53 patients had post-operative new bone formation, with a surprisingly high percentage of 94.33%. In the age of 16–20 group, there were 67 out of 83 patients had post-operative new bone formation and the proportion decreased to 80.72%. In the age of 21–25 and 26–30 group, there were 25 and 13 patients that have new bone formation, the proportion further decreased to 54.35% and 56.52%, respectively. However, in the age above 30 group, only 6 (25%) patients were found to have new bone formation (Table 2). The results are shown in Table 3.

**Table 2:** Gender and age distribution of patients (*p < 0.05).

	Male	Female	Age
Number of patients	29	200	21.5 ± 3.5
New bone formation (Yes)	26 (89.66%)	135 (67.5%)	17.5 ± 2.1*
New bone formation (No)	3 (10.34)	65 (32.5%)	24.5 ± 0.7*

**Table 3:** New bone formation in different age distributions of patients.

Age	Number of patients	Number of new bone formation	Percentage (%)
10–15	53	50	94.33
16–20	83	67	80.72
21–25	46	25	54.35
26–30	23	13	56.52
Above 30	24	6	25
Total	229	161	70.3 (average)

### The position of the new bone formed on the condyle

**Figure 1:**
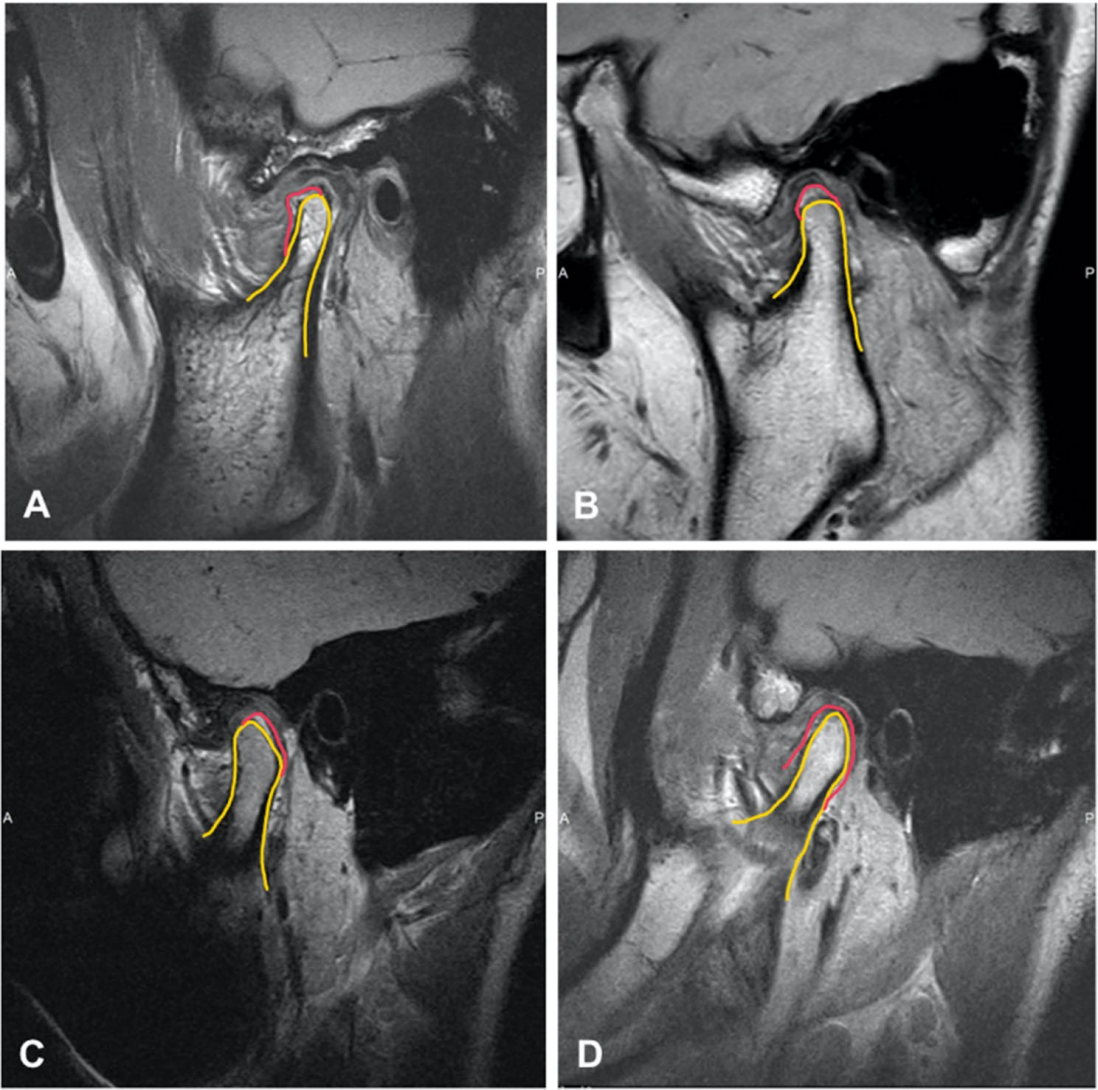
Condylar new bone formation was found on different position. (**A**) Anteriorslope of condyle. (**B**) On the top of condyle. (**C**) Posterior slope of condyle. (**D**) Around condyle (yellow line: condyle cortical bone outline; red line: new bone outline).

Through screening the MRI examinations of those patients, 161 patients of 241 joints in this study were found new bone formation located anteriorly (Fig. 1A), on the top of the condyle (Fig. 1B), posteriorly (Fig. 1C) or around the condyle (Fig. 1D).

According to this classification, there were 91 patients with 129 joints (53.53%) had new bone formation on the posterior slope of the condyle. Secondly, there were 37 patients with 54 joints (22.41%) had new bone formation on the top of the condyle. Thirdly, there were 24 patients with 33 joints (13.69%) had new bone formation around the condyle. While on the anterior slope of the condyle, there were 20 patients with 25 joints (10.37%). The results were shown in Table 4.

**Table 4:** Different position of the new bone formed on the condyle.

Position of new bone	Number of patients	Number of joints	Percentage (%)
Posterior slope of condyle	91	129	53.53
Top of condyle	37	54	22.41
Around condyle	24	33	13.69
Anterior slope of condyle	20	25	10.37
Total	172	241	100

### Typical patient case

**Figure 2:**
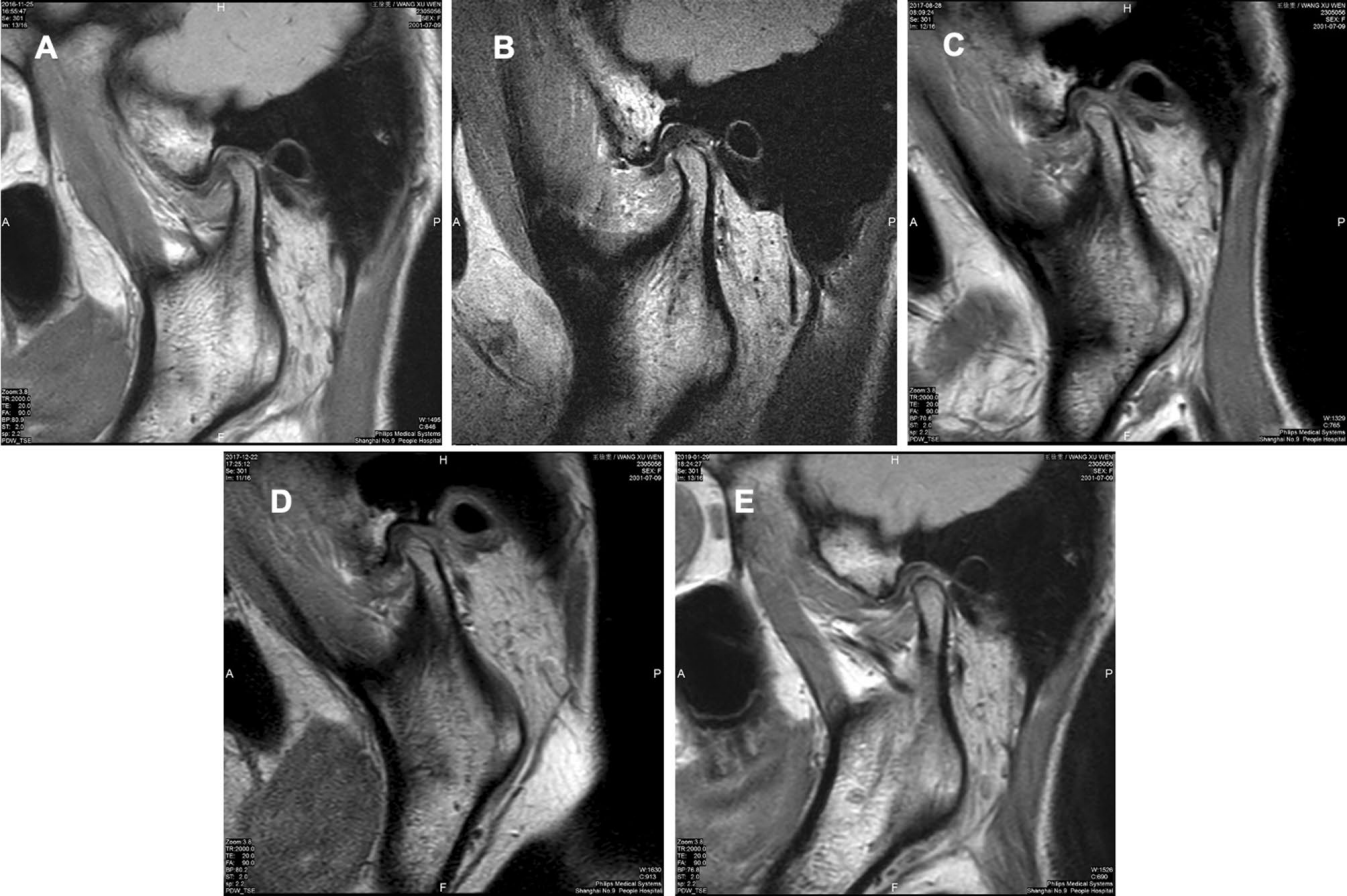
Typical case of new bone formation. (**A**) Pre-operative MRI showed TMJ ADDWoR. (**B**) Post-operative MRI showed well repositioned TMJ disc. (**C**) 6 months post-operatively, the disc was replaced and new bone was formed on the posterior slope of condyle. (**D**) 12 months post-operatively, newly formed bone reconstructed with the condyle. (**E**) 24 months post-operatively, the border between new bone and condyle vanished, the condyle was well remodeled.

A 15-year-old girl was diagnosed as TMJ ADDWoR (Fig. 2A), the patient received Yang’s arthroscopic surgery on Feb 2017 and the immediate post-operative MRI revealed satisfied disc position (Fig. 2B). 6 months after surgery, MRI showed good relationship between disc and condyle, meanwhile, a large amount of new bone was observed on the posterior slope of the condyle (Fig. 2C). 12 months post-operatively, the newly formed bone remodeling was observed from the MRI (Fig. 2D) and on the 24 months post-operative MRI, the border between new bone and condyle was vanished and the condyle reconstructed well (Fig. 2E).

## Discussion

Temporomandibular joint is composed of articular fossa, disc, condyle, joint capsule and ligament around joint. It has complex anatomical structure, fine movement and undertakes important physiological functions. At present, the most commonly used imaging examination methods for TMJ are panoramic radiographs (X-ray), CT and MRI. Different examination methods are specific and sensitive to different diseases. Among them, MRI can simultaneously image the soft and hard tissues and reflect the exudation of tissue fluid, so it is very suitable for the diagnosis of temporomandibular joint disease. It is reported that MRI is very efficient in the diagnosis of anterior disc displacement^7–9^. Compared with CBCT, MRI can also reflect the changes of condylar bone. In ADDWoR patients, condylar degenerative bone changes were observed and flattening of mandible condyle active surface was the most frequent degenerative bone change (45%)^10^. More importantly, MRI is noninvasive and more suitable for the examination of the adolescent patients than other examination methods. Therefore, MRI was selected as the evaluation criteria in this study.

TMJ ADD is a kind of disease characterized by joint clicking or noises, mandible movement dysfunction (open mouth limitation), joint pain and masticatory difficulty^11^. Among TMJ ADD patients, adolescent patients, especially female, have a greater incidence^12^. In recent years, the correlation between ADD and facial symmetry has drawn more attention. It has been reported in literature that ADD in growing patients may cause condylar deformity, decreased ramus height and mandibular length^13–16^. These results have also been verified through animal experiments, in rabbit model, unilateral ADD could cause shortening of the mandibular length and facial midline deviated to the affected side, while bilateral ADD would cause mandibular retrognathia^17,18^.

Through 3D finite element analysis, Nishio C^19^ indicated that among the patients with ADD, the anterior part of the condylar surface showed the largest stress within the first 2 min, then decreased gradually and reached a steady level. While all the other areas showed a gradually increased stress, especially the posterior area which exhibited the largest stress. Ichimiya H^20^ reported that static mechanical compression promoted osteoclast formation through up-regulation of receptor activator of nuclear factor kappa-B ligand (RANKL) expression in synovial cells. Excessive stress on the condylar head and disc synovial tissues can promote the formation of osteoclasts, leading to the degenerative changes such as osteoarthritis or condylar resorption. Thus, the change of mechanical loading on the surface of condylar head is one of the probable causes of bone resorption.

Therefore, in the growing period, the position of TMJ disc is of great significance to the development of mandible and the facial appearance. Therefore, in the early stage of the disease, timely correction of the relationship between disc and condyle can ensure the restoration of normal condyle and mandible development, which is of great significance to prevent the further progress of the disease and correct the facial appearance.

Some scholars^21,22^ have studied the bone remodeling after TMJ arthroscopic disc reposition. Liu XH et al.^22^ conducted a retrospective study on the formation of new bone in patients after arthroscopy through the study method of prognosis nomogram. The results showed that the rate of new bone formation after arthroscopy was about 50% considering the sex, age, Wilkes stage, bad oral habits (such as bruxism). Compared with the results of this study, the new bone formation rate is relatively low, which may be related to the ungrouping of different factors. However, of all the influencing factors, age had the greatest effect on the formation of new bone after operation. The younger the age, the stronger the new bone formation ability after operation. This is consistent with the results of this study.

On the other hand, it may be due to the continuous improvement of surgical techniques and perioperative management, including complete anterior disc attachment releasement, proper disc reduction (over correction), and postoperative occlusal relationship adjustment. In order to reduce the postoperative recurrence rate, during the operation, the operator will overcorrect the articular disc^6^. At this time, the articular disc is in a posterosuperior position relative to the condyle, and together with the use of postoperative occlusal splint^21^, the posterosuperior space of the joint increased^23,24^. Although the stress distribution of the condyle and articular disc needs to be further verified after reduction of the disc, the results of this study are encouraging.

In this study, there were 161 (70.3%) patients that had new bone formation after receiving the arthroscopic disc repositioning and suturing surgery, as shown in Table 2, the average age of patients with new bone formation was 17.5 ± 2.1a. In contrast, in the group without new bone formation, the average age of the patients was 24.5 ± 0.7a. There was significant difference in age between the two groups (*p < 0.05). The results indicated that after repositioning of the TMJ disc, the condyle still had a certain ability of growth, and the younger the patient was, the greater the possibility of new bone formation of condyle would have. As for the position of the new bone formation, it is clear that the posterior slope of condyle could form more new bone, which is maybe due to the increase of posterosuperior space. Meanwhile, the new bone formed in the posterior slope of condyle is helpful to improve the mandibular retrusion.

Furthermore, by analyzing the relationship between age distribution and new bone formation, we found that in the age group of 10–20 years old, the rate of patients with new bone formation was more than 80%, and the younger the age, the higher the probability of new bone formation, which is also a further verification of the above results. In terms of gender differences, the results showed that the probability of new bone formation in male patients is higher than that in female patients. However, the reason for this difference may be related to the large difference in the number of male and female patients. Therefore, more male patients should be included in the follow-up study, and the effect of gender on the formation of new bone should be studied.

There are still some limitations in this study. The amount of new bone formation is lack of quantitative analysis. Whether new bone formation is helpful for the improvement of facial symmetry after operation needs further study, which require cephalometric measurements to evaluate the improvement of facial symmetry with new bone formation.

## Conclusion

In summary, this study evaluated the post-operative condylar bone remodeling using MRI, the results indicated that the condylar bone had a strong ability of growth and remodeling after disc reposition, especially in the posterior slope of condyle, and the condylar growth ability decreased with age. Therefore, it is suggested to intervene in the early stage of TMJ ADD to restore the normal relationship between disc and condyle.
